# A Historical-Trajectories-Based Map Matching Algorithm for Container Positioning and Tracking

**DOI:** 10.3390/s22083057

**Published:** 2022-04-15

**Authors:** Wenfeng Li, Wenwen Zhang, Cong Gao

**Affiliations:** School of Transportation and Logistics Engineering, Wuhan University of Technology, Wuhan 430063, China; liwf@whut.edu.cn (W.L.); zww96@whut.edu.cn (W.Z.)

**Keywords:** container positioning and tracking, map matching, historical trajectories, path reconstruction

## Abstract

Positioning and tracking of containers is becoming an urgent demand of container transportation. Map matching algorithms have been widely applied to correct positioning errors. Because container trajectories have the characteristics of low sampling rate and missing GPS points, existing map matching algorithms based on the shortest path principle are not applicable for container positioning and tracking. To solve this problem, a historical-trajectories-based map matching algorithm (HTMM) is proposed. HTMM mines the travel time and the frequency in historical trajectories to help find the local path between two adjacent candidate points. HTMM first presents a path reconstruction method to calculate the travel time of historical trajectories on each road segment. A historical path index library based on a path tree is then developed to efficiently index historical paths. In addition, a location query and tracking method is introduced to determine the location of containers at given time. The performance of HTMM is validated on a real freight trajectory dataset. The experimental results show that HTMM has more than 3% and 5% improvement over the ST-Matching algorithm and HMM-based algorithm, respectively, at 60–300 s sampling intervals. The positioning error is reduced by half at a 60 s sampling interval.

## 1. Introduction

As the core of cargo transportation, containers need to be positioned and tracked remotely in transit during transportation. The positioning and tracking of containers is mostly realized by installing satellite positioning devices on containers, collecting and transmitting data to a remote monitoring platform [1]. Satellite positioning devices are usually powered by microbatteries. Considering the high energy consumption of positioning and communication and the long transportation cycle, the sampling rate is generally set at 2 min or more to extend the battery life [2]. The distance between two adjacent GPS points is too far and cannot accurately reflect the real path of a container [3]. Moreover, the positioning signal becomes weak or even interrupted in tunnels or under viaducts and tall buildings, which causes missing and inaccurate trajectories [4]. These problems cause blind zones in the positioning and tracking of containers, and high precision positioning cannot be achieved. Therefore, accurate positioning under scenarios of low sampling rate and missing and inaccurate trajectories is a major challenge in container transportation.

Map matching algorithms, which correct errors by matching GPS points to the most probable road, have been verified as effective for map matching of passenger transport system [5,6]. To date, there are few map matching algorithms for container transportation with low-sampling-rate trajectories. Hidden Markov model (HMM)-based map matching algorithms (such as ST-Matching algorithm [7]) have been applied to match trajectories with low sampling rates of 60–300 s [8]. The commonality of these algorithms is that they choose the shortest path between two adjacent candidate points as the local path [9]. Drivers do not choose the shortest path in many cases, which reduces the accuracy of map matching algorithms. On the one hand, container transport is accompanied by cargo handling, vehicle maintenance, high toll fees and loan guarantee, etc. [10]. Drivers are more inclined to choose familiar paths or paths with relatively low comprehensive cost rather than the shortest path. On the other hand, with the decrease in sampling rate, the number of potential matched paths between two adjacent GPS points increases. Therefore, using a shortest-path-based map matching algorithm to match low-sampling-rate trajectories of container transport can easily lead to matching errors.

The information in historical trajectories, including travel time, frequency and driving preference of drivers, is not fully utilized in current map matching algorithms. Based on this, we wonder whether potential information can be mined from historical trajectories to help map matching of container transport with low sampling rates and missing GPS points. To implement this idea, a novel historical-trajectories-based map matching algorithm (HTMM) is presented in this paper. HTMM is an improved HMM-based map matching algorithm. In HTMM, information from historical trajectories, including travel time and frequency, is mined to help determine the local path. The historical velocity, distance and heading angle are employed in the search for an optimal path. Experiments on a real freight trajectory dataset verify the effectiveness of HTMM. In general, the major contributions of this paper are as follows:

(1) A historical-trajectories-based map matching algorithm (HTMM) consisting of local candidate path selection and optimal matched path search is proposed. The local path selection utilizes the travel time and the frequency mined from historical trajectories. Historical speed is introduced to improve the transition probability in the optimal matched path search.
(2) A historical path reconstruction and index method is proposed to make full use of the historical paths. A meta path is introduced to determine the travel time of historical paths. A historical path index library based on a path tree is created to store historical information and accelerate the index speed.
(3) A location correction and query method is presented to reconstruct matched paths and calculate the location of containers. Through this method, location correction and continuous tracking of the container are realized.

The outline of this paper is as follows. Section 2 reviews the related work of container positioning and tracking technology and map matching algorithms. Section 3 introduces a detailed discussion on the proposed HTMM algorithm. Section 4 verifies the accuracy and effectiveness of HTMM through experiments and displays the experimental results. Section 5 gives the conclusions and discusses future work.

## 2. Related Work

The Internet of Things (IoT) endows containers with intelligence, which is the basis of container positioning and tracking [11]. A container equipped with an IoT system collects identity, location, temperature and other data and transmits these data to a remote monitoring platform through a cellular mobile network, low-power LAN, LEO satellite, etc. The remote monitoring platform stores and process these data and provides data services for specified scenarios, among which container positioning and tracking is most widely demanded. For this purpose, many researchers have put forward different solutions. Nie et al. [12] designed an intelligent terminal system based on LEO satellite and narrowband Internet of Things (NB-IoT) that solves the problems of high power consumption and insufficient coverage of terminals. Lu et al. [13] proposed a container multimodal transport platform based on space-based IoT that can provide container tracking and monitoring services for different carries, such as highway, railway and waterway transportation. Tang et al. [14] proposed a refrigerated container monitoring system based on a wireless sensor network in which a geographic information system is used. This system can display container location, time, temperature and humidity information on Google Maps.

In summary, the mentioned literature points out that the following two problems need to be addressed in the design and development of positioning terminals: (1) low cost and (2) low power consumption. These two problems lead to low positioning precision and incomplete tracking of containers. Do date, few researchers have come up with useful solutions.

One method to improve positioning precision is to use pseudo-range differential positioning devices with sub-meter precision; however. high cost, missing trajectories and drift cannot be avoided. Map matching technology is a good choice to improve precision and reduce cost. Besides improvement of positioning precision, on a deeper level, trajectories after map matching can be applied to advanced applications, such as logistics tracking, location prediction and path planning or recommendation [15]. Missing and low sampling rate (60 s or more) trajectories are the research object of this paper. Simple map matching algorithms, such as geometry-based [16] and topology-based [17] map matching algorithms, have higher accuracy when the sampling rate is 1–10 s but perform worse when matching low-sampling-rate trajectories.

There are two types of map matching algorithms suitable for low-sampling-rate trajectories. One is map matching based on HMM. Newson et al. [8] first applied HMM to map matching; the accuracy can reach 99% when the sampling rate is 1–30 s but gradually decreases with sampling rates over 30 s. This map matching algorithm considers various features, such as distance and direction, when calculating observation and transition probabilities. Most assume these features satisfy a certain probability distribution. Lou et al. [7] proposed the ST-Matching algorithm on this basis. The ST-Matching combines spatial and temporal features; in particular, the road speed limit is introduced into the temporal feature. The accuracy of this algorithm can reach 80% when the sampling rate is 2.5 min. Yuan et al. [9] proposed the IVMM algorithm, which considers the influence of the context GPS point to match the current GPS point. It adds an interacting voting process on the basis of ST-Matching. When the sampling rate is 1.5–6.5 min, the accuracy of IVMM is about 70%. There are many similar map matching algorithms, such as those presented in [18,19].

Map matching algorithms based on HMM assume that the local candidate path between two adjacent points follows the shortest length/time path, which is not suitable for all low-sampling-rate trajectories and dense road networks. Map matching algorithms based on local path inference that do not rely on shortest length/time path assumptions have been proposed to solve this problem. Zheng et al. [20] believe that the user’s trajectories follow certain space–time travel rules and proposed a two-stage path inference system, HRIS, driven by historical data. The first stage is local path inference, which calculates multiple possible paths between any two adjacent points. In the second stage, a dynamic programming algorithm is used to calculate the most probable path from the local paths as the optimal matched path. Banerjee et al. [21] proposed a path inference system, InferTra, that infers the complete path of a trajectory using the Gibbs sampling algorithm and the network mobility model. Wu et al. [22] proposed a completely probabilistic path inference system, STRS, that also considers the temporal and spatial probability distribution models of historical trajectories. Compared with other map matching algorithms, the accuracy of STRS is greatly improved.

In essence, these algorithms solve the most probable path through the assumed probability distribution or prior historical trajectories. However, there are some problems in solving the positioning and tracking of container transportation:

(1) Map matching algorithms based on HMM follow the shortest length/time principle, which is not appliable for missing and low-sampling-rate trajectories. Map matching algorithms based on local path inference require a large number of historical trajectories to extract path selection preference and travel law, which lead to high time complexity.
(2) With the rapid development of roads and traffic control, only using movement modes found from historical trajectories may not be suitable for the current situation.
(3) In passenger transportation systems, trajectories come from multiple drivers with different purposes, and the movement modes found from all historical trajectories cannot represent each driver’s driving preferences.
(4) Most of the above algorithms focus on passenger transportation systems, especially floating cars in an urban road network. Container transportation systems are rarely studied, but the demand for positioning and tracking of containers is considerable.

Motivated by these problems, a map matching algorithm called HTMM is developed in this paper. The main differences between this work and previous work are as follows:

(1) HTMM is a two-stage map matching algorithm based on a hidden Markov model and a local path inference method.
(2) Both historical information and real-time vehicle movement information are considered in HTMM.
(3) HTMM aims to improve positioning precision and track the location of containers in a container transportation systems.

## 3. Methodology

### 3.1. Preliminary

In this subsection, some definitions of the map matching problem will be given as follows.

**Definition** **1** (Trajectory)**.** *Trajectory*, T*, is defined as a sequence of GPS points with timestamps, i.e.,* ${T=<p}_{1}{,\;p}_{2},\cdots {,\;p}_{n}>$*. A GPS point is expressed as* $p_{i}=<lat,\;lng,\;t,\;v,\;\gamma >\;\left(1\leq i\leq n\right)$*, which consists of the latitude (* $p_{i}.lat$*), the longitude (* $p_{i}.lng$*), the velocity (* $p_{i}.v$*) and the heading angle (* $p_{i}.\gamma$*) of* $p_{i}$ *at time* $p_{i}.t$.

**Definition** **2** (Road Network)**.** *Road network is defined as a directed graph,* G={V, E}*. Node set* V *represents the set of, end or intersection of road segments. Edge set* E *represents the set of road segments that connect the nodes in* V*. The* $e_{j}\left(1\leq j\leq \left|E\right|\right)$ *in* E *has three attributes: (1) the unique number of* $e_{j}$ *(* $e_{j}.eid$*); (2) the coordinates of the start node (* $e_{j}.start$*) and the end node (* $e_{j}.end$*) of* $e_{j}$*; and (3) the length of* $e_{j}$ *(* $e_{j}.length$*)*. |E| *is the number of edges in* E.

**Definition** **3** (Candidate road segment and candidate point)**.** *Given* $p_{i}$ *and* G={V, E}*, the candidate road segments of* $p_{i}$ *are defined as* $E_{i}=\{e_{j}|d\left(p_{i}{,\;e}_{j}\right)<\delta ,\;\forall e_{j}\in E\}$*, where* $d\left(p_{i}{,\;e}_{j}\right)$ *is the projection distance from* $p_{i}$ *to* $e_{j}$*, and* δ *is the distance threshold. The candidate point of* $p_{i}$ *on* $e_{j}$ *is expressed as* $c_{i}^{j}$.

**Figure 1:**
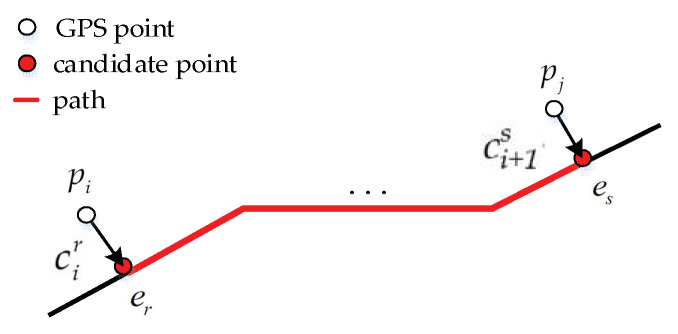
The path from the candidate point, $c_{i}^{r}$ on $e_{r}$ to the candidate point $c_{i+1}^{s}$ on $e_{s}$. $c_{i}^{r}$ is the projection point of $p_{i}$, and $c_{j}^{s}$ is the projection point of $p_{i+1}$.

**Definition** **4** (Path)**.** *A path is a sequence of road segments. As shown in* Figure 1*, the path from* $c_{i}^{r}$ *to* $c_{i+1}^{s}$ *is expressed as* $P_{r\to s}:\left\{e_{r}\to e_{r+1}\to \cdots \to e_{s}\right\}$*, in which* $c_{i}^{r}$ *is on* $e_{r}$ *and* $c_{i+1}^{s}$ *is on* $e_{s}$.

**Definition** **5** (Matched point)**.** *The candidate point on the global optimal path is the matched point. The matched point of GPS point* $p_{i}$ *on road segment* $e_{j}$ *is expressed as* $m_{i}^{j}$. $m_{i}^{j}$ *has two attributes: (1) the error of the GPS point and the matched point (* $m_{i}^{j}.error$*); (2) the distance from $m_{i}^{j}$ to the start node of* $e_{j}$ *(* $m_{i}^{j}.offset$*)*.

Due to the measurement error of the positioning device, the GPS points are not always on the road of the map. Therefore, the goal of the map matching algorithm is to find the most probable path under the given trajectory, T, and the road network, G = {V, E}.

### 3.2. Notation Description

Table 1 presents the relevant indices and variables for HTMM.

### 3.3. System Overview

**Figure 2:**
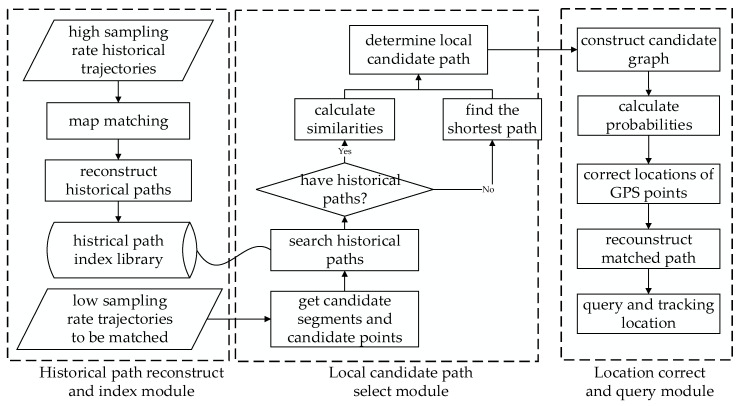
Framework of HTMM. HTMM is made up of three modules: a historical path reconstruction and index module, a local candidate path selection module, and a location correction and query module.

As shown in Figure 2, the framework of HTMM consists of three parts: the historical path reconstruction and index module, the local candidate selection module, and the location correction and query module. In the historical path reconstruction and index module, the historical trajectories with high sampling rates are matched to determine the historical paths. A path reconstruction method is proposed to generate meta paths, which record the enter time and the leave time of the historical paths. A historical path index library is built to index paths efficiently. In the local candidate path selection module, a local candidate path selection strategy is introduced to determine the path between two adjacent candidate points, in which the similarity of travel time and the frequency are calculated. In the location correction and query module, an improved matched point search method based on the HMM model is used to calculate the matched points. A location query and tracking method based on the matched result is introduced to obtain the location of the container at a given time.

### 3.4. Historical Path Reconstruction and Index Module

In this subsection, a historical path reconstruction and index method is proposed to make better use of the historical paths.

#### 3.4.1. Map Matching

The map matching algorithm based on HMM proposed by Newson et al. [8] is utilized to obtain the real paths of the historical trajectories. This algorithm has 99% accuracy when the sampling rate is under 30 s. The matched paths can be considered to represent where the containers really pass through.

#### 3.4.2. Path Reconstruction 

After map matching, two adjacent matched points may be on the same road segment or across multiple road segments, so the travel time of historical paths on each road segment are unknown. A path reconstruction method based on time and distance interpolation is proposed in this subsection. A constructed path consists of several meta paths, which are defined as follows:

**Definition** **6** (Meta Path)**.** *The meta path of road segment* $e_{j}$ *is represented as* ${MP}_{j}$*, which is associated with the enter time on* $e_{j}$ *(* ${MP}_{j}.enter\_time$*) and the leave time on* $e_{j}$ *(* ${MP}_{j}.leave\_time$*). The path* $P_{r\to s}=\left\{e_{r}\to e_{r+1}\to \cdots \to e_{s}\right\}$ *consists of a meta path sequence* $\left\{{MP}_{r},{\;MP}_{r+1},\;\cdots ,{\;MP}_{s}\right\}$.

The travel time of historical paths is calculated to reconstruct the meta path. Three situations of the location relationship of two adjacent matched points need to be considered.

**Situation** **1**: $m_{i}^{r}$ and $m_{i+1}^{r+1}$ are on the adjacent road segments $e_{r}$ and $e_{r+1}$, respectively.

**Figure 3:**
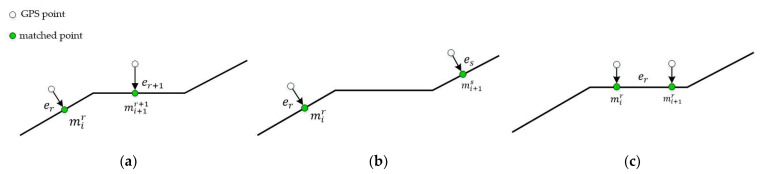
Three situations of the location relationship of adjacent two matched points: (**a**) $m_{i}^{r}$ and $m_{i+1}^{r+1}$ are on adjacent road segments; (**b**) $m_{i}^{r}$ and $m_{i+1}^{s}$ are separated by multiple road segments; (**c**) $m_{i}^{r}$ and $m_{i+1}^{r}$ are on the same road segment.

As shown in Figure 3a, the leave time of ${MP}_{r}$ is equal to the enter time of ${MP}_{r+1}$, i.e., ${MP}_{r}{.leave\_time=MP}_{r+1}.enter\_time$. It is supposed that the carrier of the container travels at a constant speed. ${MP}_{r}.leave\_time$ is calculated as:

(1) $$\left\{\begin{array}{l} {MP}_{r}{.enter\_time=p}_{i}.t+\left(p_{i+1}.t-p_{i}.t\right)\times \frac{dist\left(m_{i}^{r}{,\;e}_{r}.end\right)}{len\left(m_{i}^{r}{,\;m}_{i+1}^{r+1}\right)}\\ dist\left(m_{i}^{r}{,\;e}_{r}.end\right){=e}_{r}.length-m_{i}^{r}.offset\;\\ len\left(m_{i}^{r}{,\;m}_{i+1}^{r+1}\right)=dist\left(m_{i}^{r}{,\;e}_{r}.end\right)+m_{i+1}^{r+1}.offset\; \end{array}\right.$$

where ${dist(m}_{i}^{r},e_{r}.end)$ is the distance between $m_{i}^{r}$ and $e_{r}.end$, and ${len(m}_{i}^{r},m_{i+1}^{r+1})$ is the path length between $m_{i}^{r}$ and $m_{i+1}^{r+1}$.

**Situation** **2**: $m_{i}^{r}$ and $m_{i+1}^{s}$ are separated by multiple road segments.

As shown in Figure 3b, other road segments between $e_{r}$ an $e_{s}$ have no matched points. The enter time and leave time of the meta paths of these road segments are calculated by time and distance interpolation, as formulated in Equation (2):

(2) $$\left\{\begin{array}{l} {MP}_{r}.leave\_time=p_{i}.t+\left(p_{i+1}.t-p_{i}.t\right)\times \frac{dist\left(m_{i}^{r}{,\;e}_{r}.end\right)}{len\left(m_{i}^{r}{,\;m}_{i+1}^{s}\right)}\\ {MP}_{r+1}{.leave\_time=MP}_{r}.leave\_time+\left(p_{i+1}.t-p_{i}.t\right)\times \frac{e_{r+1}.length}{len\left(m_{i}^{r}{,\;m}_{i+1}^{s}\right)}\\ {MP}_{s}{.leave\_time=MP}_{s-1}.leave\_time+\left(p_{i+1}.t-p_{i}.t\right)\times \frac{e_{s}.length}{len\left(m_{i}^{r}{,\;m}_{i+1}^{s}\right)} \end{array}\right.$$

**Situation** **3**: $m_{i}^{r}$ and $m_{i+1}^{r}$ are on the same road segment, $e_{r}$.

As shown in Figure 3c, the matched point, $m_{i}^{r}$, does not take new information for the calculation of its travel time. The enter time and the leave time of ${MP}_{r}$ should be calculated according to the next matched point that is not on $e_{r}$.

For the meta path of the road segment on which the first (or last) matched point is located, the enter time (or the leave time) is equal to the timestamp of the first (or the last) GPS point.

#### 3.4.3. Historical Path Indexing

To accelerate the indexing speed of the historical paths, a historical path index structure called an HP-tree is built based on a path tree. The HP-tree is an improvement on FP-tree proposed by Huang et al. [23,24]. The HP-tree has the following characteristics:

(1) Each node of the HP-tree represents a road segment, among which the root node is the start road segment of the historical path (or sub path) and the leaf node is the end road segment of the historical path (or sub path).
(2) The child node of a node represents the next road segment to which it directly connects.
(3) All HP-trees make up an HP-forest to represent all historical paths and sub paths.

Each node in the HP-tree is associated with: (1) sid, which represents the road segment number and equals to eid of the node; (2) cnt, which represents the number of times the node is passed through; and (3) stime, which represents the travel time of historical paths on the node. stime is represented in the form of a dictionary, the key of which is the trajectory id, tid, the value of which is a list of travel times of historical paths on the road segment.

Each HP-tree is associated with: (1) the root node, root and (2) the relational list, idx_list. idx_list is represented in the form of a dictionary, the key of which is the road segment number, sid, the value of which is a list of nodes with the same sid.

**Figure 4:**
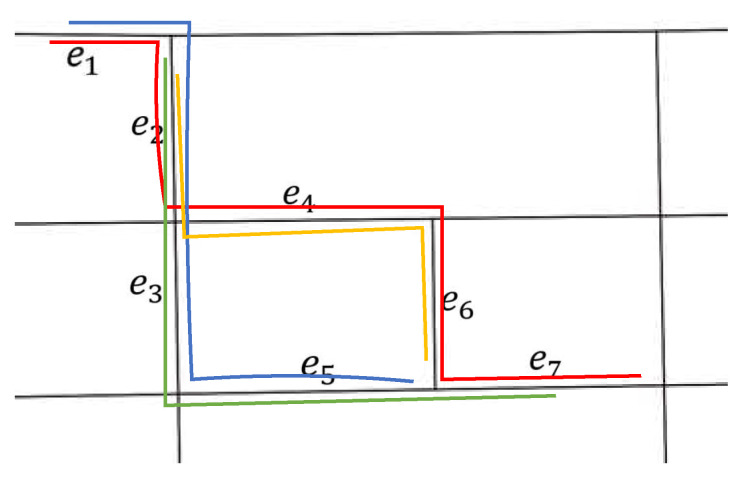
Four trajectories in the road network: $P_{1\to 7}{\;=\;\{e}_{1}\to e_{2}\to e_{4}\to e_{6}\to e_{7}\}$ (red line), $P_{1\to 5}{\;=\;\{e}_{1}\to e_{2}\to e_{3}\to e_{5}\}$ (blue line), $P_{2\to 7}{\;=\;\{e}_{2}\to e_{3}\to e_{5}\to e_{7}\}$ (green line) and $P_{2\to 6}{\;=\;\{e}_{2}\to e_{4}\to e_{6}\}$ (yellow line).

**Figure 5:**
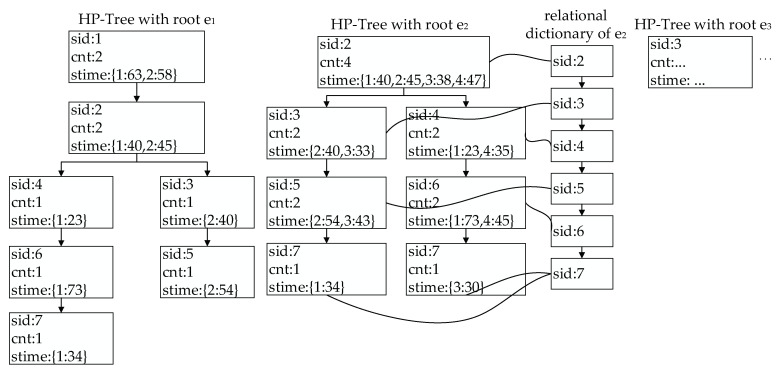
Illustration of HP-forest. The HP-tree with root $e_{1}$ stores two historical paths: ${\{e}_{1}\to e_{2}\to e_{4}\to e_{6}\to e_{7}\}$ and ${\{e}_{1}\to e_{2}\to e_{3}\to e_{5}\}$. The HP-tree with root $e_{2}$ stores two historical paths: ${\{e}_{2}\to e_{3}\to e_{5}\to e_{7}\}$ and ${\{e}_{2}\to e_{4}\to e_{6}\}$.

As shown in Figure 4, there are 4 historical paths: $P_{1\to 7}{\;=\{e}_{1}\to e_{2}\to e_{4}\to e_{6}\to e_{7}\}$, $P_{1\to 5}{\;=\{e}_{1}\to e_{2}\to e_{3}\to e_{5}\}$, $P_{2\to 7}{\;=\{e}_{2}\to e_{3}\to e_{5}\to e_{7}\}$ and $P_{2\to 6}{\;=\{e}_{2}\to e_{4}\to e_{6}\}$. Seven HP-trees with $e_{1}-e_{7}$ as root nodes can be created. Only the index structure of two of them are shown in Figure 5. With the help of an HP-tree, historical paths between given start and end road segments can be efficiently searched. For example, to search all the historical paths between $e_{2}$ and $e_{7}$, the HP-tree with root node $e_{2}$ is first found; then, the relational list, idx_list, with key $e_{7}.eid$ is searched. Each node in idx_list is traversed, and its parent node is recursively traversed until the root node is reached. The historical paths are obtained by outputting these nodes in reverse order. Therefore, the historical paths are ${\{e}_{2}\to e_{3}\to e_{5}\to e_{7}\}$ and ${\{e}_{2}\to e_{4}\to e_{6}\to e_{7}\}$. cnt and stime of each road segment in historical paths can be obtained at the same time.

### 3.5. Local Candidate Path Selection Module

**Figure 6:**
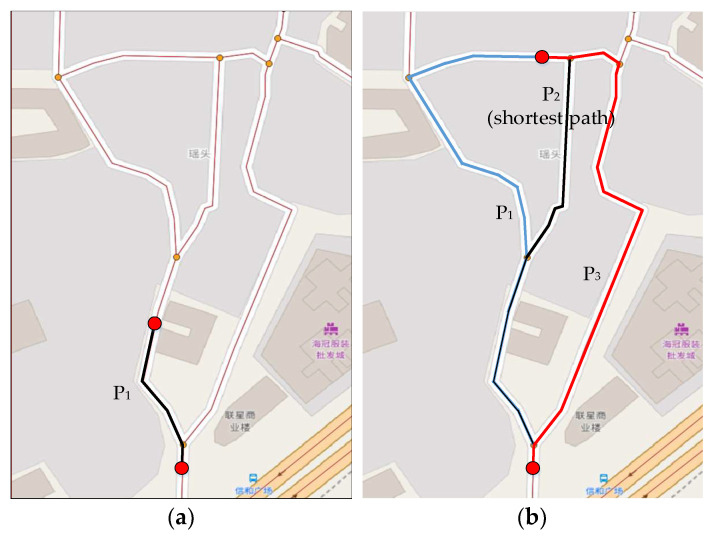
Possible paths from candidate point $c_{i}^{r}$ to $c_{i+1}^{s}$: (**a**) for high-sampling-rate trajectories, both the shortest path and the local candidate path is $P_{1}$; (**b**) for low-sampling-rate trajectories, $P_{2}$ is the shortest path, but the local candidate path may be $P_{1}$, $P_{2}$ or $P_{3}$.

For the missing and low-sampling-rate trajectories of containers, the candidate points of two adjacent GPS points may not be in the same road segment or adjacent road segments. There may be multiple paths to select between two adjacent GPS points. It is assumed that the path between two adjacent GPS points follows the shortest path principle in the map matching algorithm based on HMM and several improved algorithms. In Figure 6a, only one path between two candidate points, $c_{i}^{r}$ and $c_{i+1}^{s}$, of high-sampling-rate trajectories is found, which is the shortest path. However, in Figure 6b, 3 possible paths are found between $c_{i}^{r}$ and $c_{i+1}^{s}$ of low-sampling-rate trajectories. Based on the shortest path principle, $P_{2}$ is selected as the local candidate path. However, drivers may choose $P_{1}$ or $P_{3}$ under different traffic conditions and based on personal preferences.

A local candidate path selection strategy that takes the travel time and the frequency of historical paths into consideration is proposed in this paper and is elaborated in the following section.

#### 3.5.1. Possible Paths Search

To find all the possible paths between two adjacent candidate points, the directed graph, G={V, E}; the end node of $e_{r}$; and the start node of $e_{s}$ are found and viewed as the source node and the target node, respectively. The steps of the search strategy are as follows:

(1) Search the historical paths between $e_{r}.end$ and $e_{s}.start$ in the HP-Forest.;
(2) If no historical path is found, the shortest path between $e_{r}.end$ and $e_{s}.start$ is selected as the local candidate path;
(3) If only one historical path is found, then it is the local candidate path. If multiple historical paths are found, then add all these historical paths to the possible path set.

#### 3.5.2. Similarity Calculation

(1) Travel Time Estimation

Considering road conditions and driving habits of drivers, the shortest path is not necessarily the best choice. It is assumed that the driver is more likely to choose the path with a travel time is close to that of the current trajectory. Road-segment-based and path-based methods are commonly used for path travel time estimation [22]. The travel time of a road segment is obtained according to the estimated average speed in the road-segment-based method. The travel time of a path is the sum of the travel time of all the road segments. However, it cannot be used very effectively when speed changes suddenly at crossroads or speed is not available. In a path-based method, the average time of paths is calculated by retrieving the same paths form the historical paths. Although speed estimation is avoided, there are rarely two identical paths in the historical paths.

A travel time estimation method combining a road-segment-based method and a path-based method is proposed in this paper. Because the possible paths between two adjacent candidate points are the sub paths of the historical paths, the problem with the path-based method is avoided. The travel time estimation of road segments is obtained by calculating the average travel time of the corresponding road segment from multiple identical possible paths. The estimated travel time of the path is equal to the sum of estimated travel time of these road segments.

The possible path set between $c_{i}^{r}$ and $c_{i+1}^{s}$ is represented as $CP\;=\;\left\{{CP}_{1}{,\;CP}_{2},\;\ldots {,\;CP}_{l}\right\}$. Assume the historical trajectories that match ${CP}_{k}=\left\{e_{r}{,\;e}_{r+1},\;\ldots {,\;e}_{s}\right\}\left(k=\;1,\;2,\;\cdots ,\;l\right)$ are $T_{1}{,\;T}_{2},\;\cdots {,\;T}_{l}$. The travel time of $T_{k}$ on each road segment is represented as $t_{r}^{k}{,\;t}_{r+1}^{k},\;\ldots {,\;t}_{s}^{k}$. The travel time of the intermediate road segments, $e_{r+1},\;\ldots {,\;e}_{s-1}$, that are passed through completely is formulated as:

(3) $$\left\{\begin{array}{l} {\bar{t}}_{r+1}=\overset{l}{\underset{k=1}{\sum }}t_{r+1}^{k}/l\\ {\bar{t}}_{r+2}=\overset{l}{\underset{k=1}{\sum }}t_{r+2}^{k}/l\\ \ldots \\ {\bar{t}}_{s-1}=\overset{l}{\underset{k=1}{\sum }}t_{s-1}^{k}/l \end{array}\right.$$

For $e_{r}$ and $e_{s}$, the location of the matched point on the road segment needs to be considered when calculating the travel time. The calculation formula is:

(4) $$\left\{\begin{array}{l} {\bar{t}}_{r}=(\overset{l}{\underset{k=1}{\sum }}t_{r}^{k}{/T}_{k}{.m}_{i}^{r}.offset)/l\\ {\bar{t}}_{s}=(\overset{l}{\underset{k=1}{\sum }}t_{s}^{k}{/T}_{k}{.m}_{i+1}^{s}.offset)/l \end{array}\right.$$

Therefore, the similarity of the time difference between $c_{i}^{r}$, $c_{i+1}^{s}$ and the estimated travel time of ${CP}_{k}$ is:

(5) $$S_{k}^{time}=\frac{{(p}_{i+1}.t-p_{i}.t)-\sum_{j=r}^{s}{\bar{t}}_{j}}{{(p}_{i+1}.t-p_{i}.t)}$$

(2) Frequency Calculation

Path frequency is the second consideration when selecting the local candidate path. Through the observation of drivers, Gao et al. [25] found that about 60% of driving paths are repeated for at least 40 days.

The number of times all road segments in ${CP}_{k}$ are passed through by historical trajectories is represented as $c_{r}^{k}{,\;c}_{r+1}^{k}\cdots {,\;c}_{s}^{k}$. It is found that the number of times that ${CP}_{k}$ is passed through is equal to $c_{s}^{k}$.

Therefore, the frequency of ${CP}_{k}$ is:

(6) $$F_{k}=\frac{c_{s}^{k}}{\sum_{k=1}^{l}c_{s}^{k}}$$

(3) Path Selection

The travel time similarities and the frequencies of all possible paths in CP are calculated by integrating the weights, $w_{time}$ and $w_{fre}$. The comprehensive score, $S_{k}$, is formulated as:

(7) $$S_{k}{\;=w}_{time}\times S_{k}^{time}+w_{\mathrm{fre}}\times F_{k}$$

The local candidate path is the most similar with the largest $S_{k}$.

### 3.6. Location Correction and Query Module

A matched point search strategy is proposed in this subsection. More specifically, the matched point is the corrected GPS point. Because the GPS points are discrete, the location of a GPS point cannot be known every time. Therefore, a method of location query of GPS points is introduced.

#### 3.6.1. Optimal Matched Point Search

**Figure 7:**
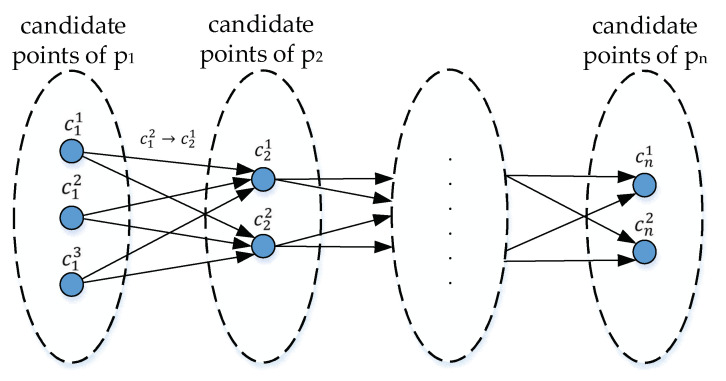
Candidate graph composed of candidate points and local candidate paths. Each candidate point has an observation probability, and the probability from the previous candidate point to the current candidate point is expressed as a transition probability.

As shown in Figure 7, each GPS point has multiple candidate points, so there are multiple local candidate paths between two adjacent GPS points. In order to determine which candidate point the GPS point matches to, the probabilities of all local candidate paths are calculated.

(1) Observation Probability

The observation probability represents the possibility that $p_{i}$ matches to $c_{i}^{r}$. The distance and the heading angle are taken into consideration; then, the observation probability $p_{o}\left(c_{i}^{r}\right)$ is formulated as:

(8) $$p_{o}\left(c_{i}^{r}\right)=\frac{1}{\sqrt{2\pi }\sigma }e^{-\frac{{dist(p}_{i}{,\;c}_{i}^{r})}{2\sigma^{2}}}\times {\lambda e}^{-\lambda \theta }$$

where $dist\left(p_{i}{,\;c}_{i}^{r}\right)$ is the distance between $p_{i}$ and $c_{i}^{r}$ and obeys the normal distribution with mean zero and variance σ. θ is the angle between the driving direction and the direction of the road segment and obeys exponential distribution with rate parameter λ.

(2) Transition Probability

The transition probability represents the possibility that the next path of $e_{r}$ is $e_{s}$. In most map matching algorithms based on HMM, it is supposed that the transition probability is associated with the length difference between the length of a local candidate path and the distance between $p_{i}$ and $p_{i+1}$. As a result, the output tends to be the shorter path. In fact, the longer paths are filtered in the local candidate path selection module of the system, so only average velocity is considered.

It is assumed that one of the local candidate paths from $c_{i}^{r}$ to $c_{i+1}^{s}$ is $\left\{e_{r}{,\;e}_{r+1},\;\cdots {,\;e}_{s}\right\}$ because the travel time of the current trajectory on each road segment is unknown, and only the average speed can be calculated, as shown in Equation (9):

(9) $${\bar{v}}_{i\to i+1}=\frac{\sum_{j=r}^{s}e_{j}.length}{{\Delta t}_{i\to i+1}}$$

where ${\Delta t}_{i\to i+1}$ is the time difference between $p_{i}$ and $p_{i+1}$.

The ST-Matching algorithm [7] calculates velocity similarity by comparing the difference between the speed limit and the average velocity. Due to the velocity change and waiting time caused by road traffic congestion, the velocity is always much lower than the speed limit. In addition, it is difficult to obtain real-time traffic flow information. Therefore, HTMM calculates velocity similarity by comparing the difference between the historical velocity and the average velocity. The historical velocity of the road segment of the local candidate path is:

(10) $$v_{j}=\frac{e_{j}.length}{{\bar{t}}_{j}}\left(j=r,\;r+1,\;\cdots ,\;s\right)$$

Therefore, the transition probability is:

(11) $$p_{t}\left(c_{i}^{r}\to c_{i+1}^{s}\right)=\frac{\sum_{j=r}^{s}v_{j}\times {\bar{v}}_{i\to i+1}}{\sqrt{\sum_{j=r}^{s}{\left(v_{j}\right)}^{2}}\sqrt{\sum_{j=r}^{s}{\left({\bar{v}}_{i\to i+1}\right)}^{2}}}$$

The optimal matched points are denoted as $\left\{m_{1}^{r}\to m_{2}^{r+1}\to \cdots \to m_{n}^{s}\right\}$, which consists of the real path. From the first GPS point, the union probability is calculated recursively until the last GPS point is reached. The union probability is the product of the observation probability and the transition probability. The Viterbi algorithm is used to obtain the solution.

#### 3.6.2. Location Query and Tracking

In order to achieve more accurate query of cargo shippers and carriers on the locations of containers, a container location query and tracking method based on matching results is proposed.

Given time t, the location (*p*′) of the container is obtained with the following steps:

(1) Reconstruct the matched path as meta paths to obtain the enter time and the leave time according to the method in Section 3.4.2. The meta path is represented as $\left({MP}_{1}{,\;MP}_{2},\;\cdots \right)$.
(2) Find the meta path ($MP_{j}$) where the container is located, which satisfies:
  (12) $${MP}_{j}.enter\_time\leq p^{\prime }.t\leq {MP}_{j}.leave\_time$$
  so that the container is on $e_{j}$.
(3) Obtain the latitude and longitude of *p*^′^ according to the interpolation of time and average velocity, as shown in Equation (13):
  (13) $$\left\{\begin{array}{l} p^{\prime }{.lat=e}_{j}.start.lat+\frac{|t-{MP}_{j}.enter\_time|\times \bar{v}}{e_{j}.length}\times \left(e_{j}.end.lat-e_{j}.start.lat\right)\\ p^{\prime }{.lng=e}_{j}.start.lng+\frac{|t-{MP}_{j}.enter\_time|\times \bar{v}}{e_{j}.length}\times \left(e_{j}.end.lng-e_{j}.start.lng\right) \end{array}\right.$$
  where $\bar{v}$ represents the average velocity of the GPS point.

The reconstructed path can be added to the historical index library again, which reinforces the positioning and tracking of later GPS points.

## 4. Experiments

In this section, the effectiveness of HTMM is verified on a real trajectory dataset. HTMM is compared with two baseline algorithms by evaluating the matching accuracy and the matching time. The matched results in two typical situations are visualized to illustrate the improvement of HTMM. The positioning errors before and after location correction are calculated to show the effect of map matching in reducing positioning error.

### 4.1. Trajectory Dataset and Road Network

**Figure 8:**
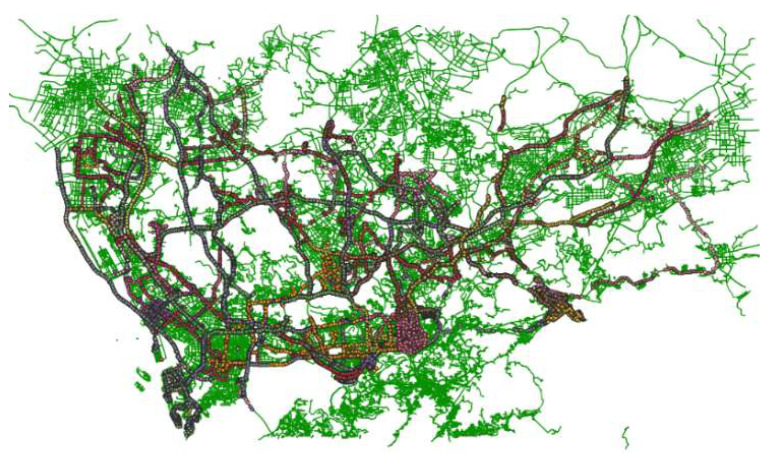
Distribution of the GPS trajectories. The green background is the road network, and other colored lines are trajectories.

The experimental dataset comes from the real-time GPS positioning records of 15000 trucks of a freight company in Shenzhen and surrounding cities. The places of departure and destination include container terminals, logistics parks, ports, etc., as shown in Figure 8.

**Figure 9:**
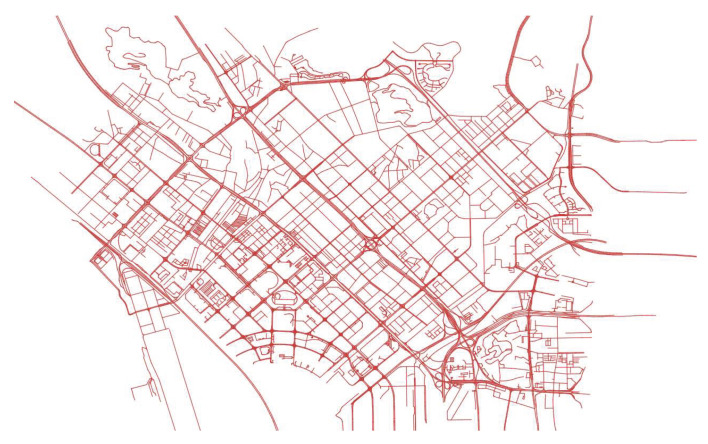
Road network of the region. The network contains 17,108 nodes and 18,892 edges.

HTMM is performed on a region whose latitude and longitude range is (22.5424, 113.8489, 22.5966, 113.9257). The road network downloaded from Open Street Map is contains 17,108 nodes and 18,892 edges, as shown in Figure 9.

**Figure 10:**
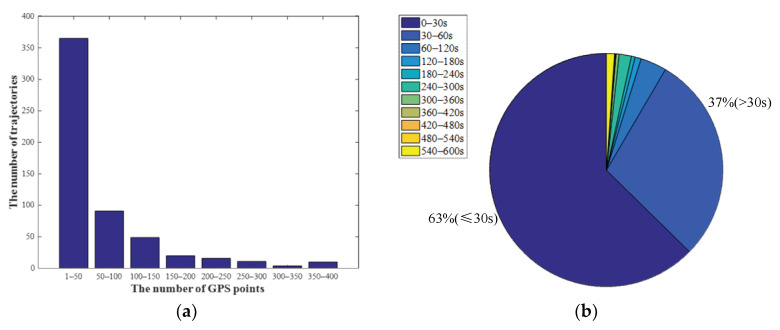
Description of the trajectory dataset: (**a**) number of GPS points of trajectories; (**b**) sampling rate of GPS points.

Before the trajectory dataset is used for map matching, the noise and redundant GPS points are filtered, and the long trajectories are segmented. The number of GPS points of each trajectory is displayed in Figure 10a, and the sampling rate of each GPS point is displayed in Figure 10b. Most trajectories have fewer than 50 GPS points, and the sampling rates of 63% of GPS points are higher than 30 s.

### 4.2. Experimental Settings

**Ground Truth**: A total of 566 high-sampling-rate historical trajectories with sampling intervals less than 30 s are selected to match HMM-based map matching algorithm to obtain the real paths as ground truth. Then, these real paths are used to reconstruct meta paths and build a historical path index library.

**Baselines**: An HMM-based map matching algorithm [8] and the ST-Matching algorithm [7] are used as the comparison algorithms to verify the effectiveness of HTMM.

**Evaluation Criteria**: Matching accuracy and matching time are chosen to evaluate HTMM. The matching time is measured by the running time of the program, which does not include the time to build the historical path index library and load the road network data. The matching accuracy is measured by the proportion of the number of correctly matched road segments to the total number of road segments of trajectories, as shown in Equation (14):

(14) $$A_{N}=\frac{N_{c}}{N_{t}}$$

where $N_{c}$ represents the number of correctly matched road segments, and $N_{t}$ represents the number of total road segments.

Test Data: A total of 100 high-sampling-rate trajectories are selected to generate test data, which are first matched to the road network to obtain the real paths as ground truth, and then down-sampled at 60 s, 120 s, 180 s, 240 s, 300 s, 360 s, 420 s, 480 s and 600 s intervals.

### 4.3. Experimental Results

#### 4.3.1. Comparison of Three Map Matching Algorithms

**Figure 11:**
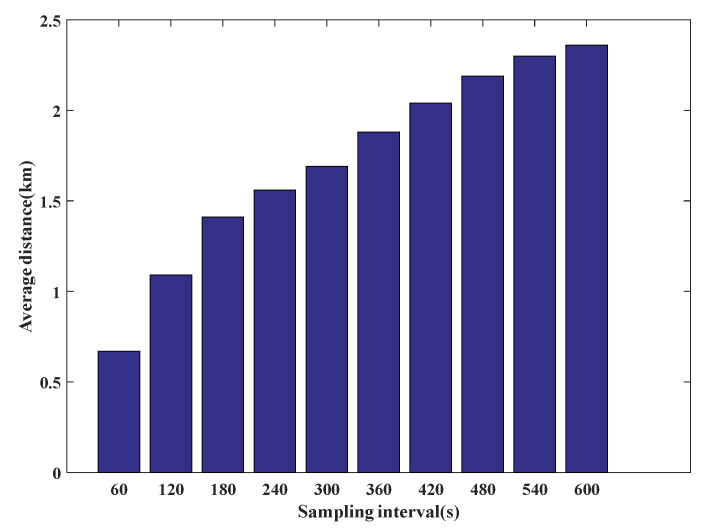
The average distance between two adjacent GPS points at different sampling rates.

A change in sampling rate leads to a change of the distance between two adjacent GPS points. Figure 11 shows the average distance between two adjacent GPS points of the test data under different sampling rates. The average distance is 0.67 km when the sampling rate is 60 s but reaches 2.36 km when the sampling rate is 600 s. This introduces problems such as matching interruption and matching error and reduces the matching accuracy.

**Figure 12:**
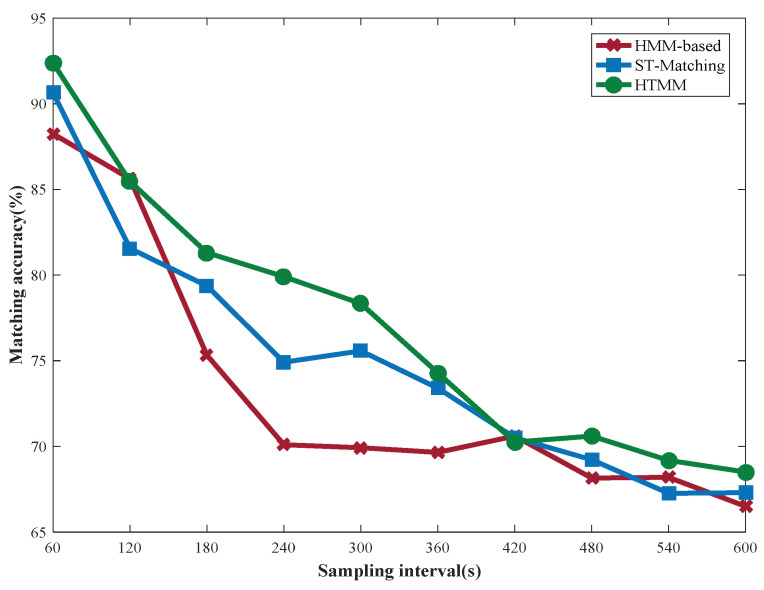
Matching accuracy of three algorithms at different sampling rates.

As shown in Figure 12, the matching accuracy of the three algorithms gradually decreases as the sampling rate decreases. It is clear that HTMM outperforms the HMM-based and ST-Matching algorithms when the sampling rate ranges from 60 s to 300 s. This result may be due to the fact that the paths between adjacent GPS points do not conform to the shortest path principle. This also proves that it is feasible to select local paths according to historical information. However, the accuracy of HTMM drops sharply when the sampling rate ranges from 360 s to 600 s. The main reason for this is that as the path length increases, there are more historical paths to choose from. Drivers also make choices according to the current situation, not just relying on historical experience. In this case, the current information of GPS points is not fully utilized by the HTMM algorithm.

**Figure 13:**
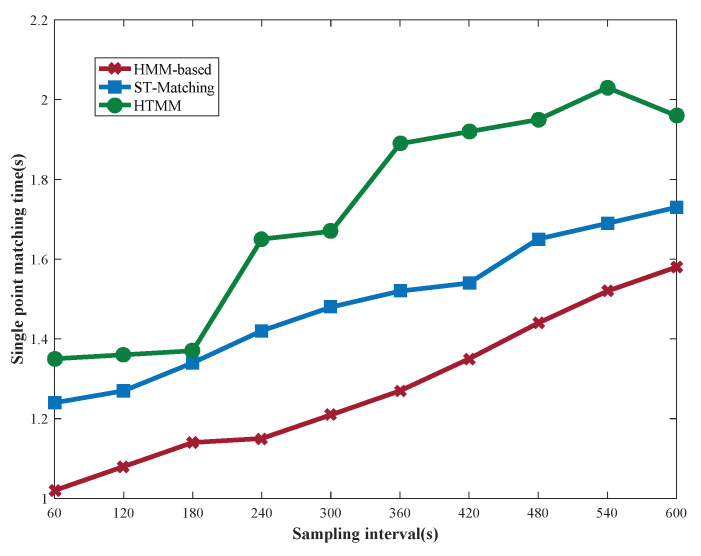
Matching time of three algorithms at different sampling rates.

As shown in Figure 13, with an increase in algorithm complexity, the matching time is increases, so HTMM is inferior to the other two algorithms. Besides the search of global optimal path, the time cost of HTMM is mainly in the calculation of multiple candidate road segments and the retrieval of the historical index library. In particular, the time cost of HTMM increases significantly with a decrease in sampling rate. However, the local candidate path is determined before searching the global matched path. Although the global optimal solution may not be obtained, this operation reduces time cost. It is acceptable to sacrifice time cost for improved matching accuracy.

#### 4.3.2. Visual Analysis of Typical Situations

Two typical matched paths are selected from the matched results for visual analysis.

(1) Detours

**Figure 14:**
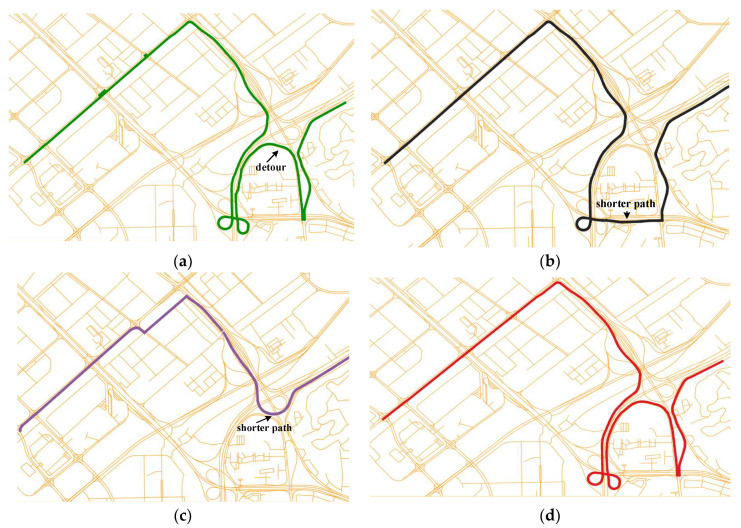
Comparison of matched paths of HTMM and ST-Matching: (**a**) sampling rate of 30 s (real path); (**b**) sampling rate of 60 s (ST-Matching); (**c**) sampling rate of 120–300 s (ST-Matching); (**d**) sampling rate of 120–300 s (HTMM).

As shown in Figure 14, there are many detours in the real path represented by the green line. After using the ST-Matching algorithm, the detour is replaced by a shorter path when the sampling rate is 60 s. The result become worse when the sampling rate is 120–300 s. This is the result of selecting the shortest path. It is found that the travel time of detours and the shortest paths is very different, so HTMM solves this problem well by calculating the travel time. However, HTMM produces errors in the selection of main roads and auxiliary roads.

(2) Multiple Optimal Paths

**Figure 15:**
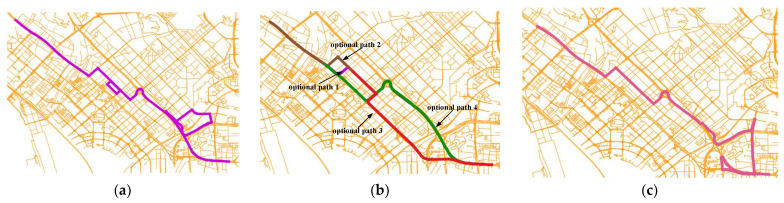
Comparison of matched paths of HTMM and ST-Matching: (**a**) sampling rate of 30 s (real path); (**b**) sampling rate of 60–300 s (ST-Matching); (**c**) sampling rate of 60–120 s (HTMM).

As shown in Figure 15, different sampling rate trajectories obtain different matched paths after using the ST-Matching algorithm. This is because the length and direction angle of these optional paths are similar, so the ST-Matching algorithm does not know which one to select. HTMM solves this problem by adding the frequency of the historical paths as the measurement of path selection. It is believed that the result is reliable if the historical path index library is personalized and large enough.

#### 4.3.3. Positioning Error Analysis

In order to verify the effect of HTMM on positioning error correction, the GPS points eliminated by down sampling are taken as the points that need to query the locations. The real locations of these GPS points are known. The meta paths of the matched path are reconstructed to acquire the enter time and the leave time; then, the corrected locations are calculated according to the method introduced in Section 3.4.2. The measurement error of the GPS point is the distance difference between the observation location and its location in the historical real path, the average of which is 15.06 ± 5.57 m. The positioning error is the distance difference between the corrected location and its location in the historical real path, as shown in Table 2. The positioning error is reduced by nearly half when the sampling rate is 60 s.

HTMM can eliminate the error perpendicular to roads. However, the error parallel to roads, which is caused by change in speed and other factors, cannot be eliminated.

## 5. Conclusions

In this paper, a novel map matching algorithm called HTMM is proposed. HTMM is the first map matching algorithm for container positioning and tracking with missing and low-sampling-rate trajectories. Compared with other methods that improve positioning precision at the hardware level, HTMM has a significant cost advantage.

The biggest innovation of HTMM is to propose a local path selection strategy that utilizes travel time and the frequency mined from historical paths. A path reconstruction method is introduced to determine the travel time of historical paths on each road segment before a historical path index library is built. A historical path index library is developed to accelerate the index speed when historical paths are searched in HTMM. Finally, to realize the query and tracking of containers, a location query and tracking method is presented by road segment retrieval and time–distance interpolation.

After verification on a real trajectory dataset, the matching accuracy of HTMM is proven to be better than that of an HMM-based map matching algorithm and the ST-Matching algorithm, especially when the sampling interval ranges from 30 s to 300 s. The matching accuracy is not improved much when the sampling interval is higher than 300 s. We infer that this is because with changing traffic conditions, the choice of drivers at each intersection between two adjacent GPS points is not necessarily the same every time. The longer the distance between two adjacent GPS points, the greater the diversity. In this case, HTMM only selects one complete historical path with the largest score as the local candidate path.

In the future work, we will further optimize the local candidate path selection strategy. One method is to segment the longer path on the basis of intersections and select a local candidate path for each partial path. Another method is to consider more real-time traffic information to improve the Markov decision process. In addition to this work, we will conduct experiments on a larger trajectory dataset and road network to improve the generalization of HTMM. HTMM will be deployed in online map matching combining with location query and tracking to realize the real-time positioning and tracking of containers, which will greatly improve the positioning precision and safety of container transportation.

## Figures and Tables

**Table 1:** Indices and variables for HTMM.

Type	Name	Meaning
Indices	i	Index of GPS points
	j, r, s	Index of edges
	k	Index of candidate path
Variables	$p_{i}$	The ith GPS point
	$p_{i}.lat$ $,\;p_{i}.lng$	The latitude and longitude of the ith GPS point
	$p_{i}.t$	The timestamp of the ith GPS point
	$p_{i}.v$	The velocity of the ith GPS point
	$p_{i}.\gamma$	The heading angle of the ith GPS point
	$e_{j}$	The jth edge
	$e_{j}.eid$	$\mathrm{The}\;\mathrm{number}\;\mathrm{of}\;e_{j}$
	$e_{j}.start$ $,\;e_{j}.end$	$\mathrm{The}\;\mathrm{start}\;\mathrm{and}\;\mathrm{end}\;\mathrm{node}\;\mathrm{of}\;e_{j}$
	$e_{j}.length$	$\mathrm{The}\;length\;\mathrm{of}\;e_{j}$
	$c_{i}^{j}$	$\mathrm{The}\;\mathrm{candidate}\;\mathrm{point}\;\mathrm{of}\;p_{i}$ $\;\mathrm{on}\;e_{j}$
	$P_{r\to s}$	$\mathrm{The}\;\mathrm{path}\;\mathrm{from}\;e_{r}$ $\;\mathrm{to}\;e_{s}$
	$m_{i}^{j}$	$\mathrm{The}\;\mathrm{matched}\;\mathrm{point}\;\mathrm{of}\;p_{i}$
	$m_{i}^{j}.error$	$\mathrm{The}\;\mathrm{distance}\;\mathrm{from}\;p_{i}$ $\;\mathrm{to}\;m_{i}$
	$m_{i}^{j}.offset$	$\mathrm{The}\;\mathrm{distance}\;\mathrm{from}\;m_{i}$ $\;\mathrm{to}\;e_{j}.start$
	${MP}_{j}^{k}$	$\mathrm{The}\;\mathrm{meta}\;\mathrm{path}\;\mathrm{of}\;T_{k}$ $\;\mathrm{on}\;e_{j}$
	${MP}_{j}^{k}.enter\_time$(leave_time)	$\mathrm{The}\;\mathrm{enter}\;\mathrm{time}\;(\mathrm{or}\;\mathrm{leave}\;\mathrm{time})\;\mathrm{of}\;{MP}_{j}^{k}$
	l	The number of candidate paths
	${CP}_{k}$	The kth candidate path
	$T_{k}$	The kth trajectory
	$t_{j}^{k}$	$\mathrm{The}\;\mathrm{travel}\;\mathrm{time}\;\mathrm{of}\;T_{k}$ $\;\mathrm{on}\;e_{j}$
	${\bar{t}}_{j}$	$\mathrm{The}\;\mathrm{estimated}\;\mathrm{travel}\;\mathrm{time}\;\mathrm{of}\;e_{j}$
	$S_{k}^{time}$	$\mathrm{The}\;\mathrm{similarity}\;\mathrm{of}\;{CP}_{k}$ on travel time
	$c_{j}^{k}$	$\mathrm{The}\;\mathrm{number}\;\mathrm{of}\;\mathrm{times}\;\mathrm{of}\;{CP}_{k}$ $\;\mathrm{on}\;e_{j}$
	$F_{k}$	$\mathrm{The}\;\mathrm{frequency}\;\mathrm{of}\;{CP}_{k}$
	$S_{k}$	$\mathrm{The}\;\mathrm{total}\;\mathrm{similarity}\;\mathrm{of}\;{CP}_{k}$
	$p_{o}{(c}_{i}^{r})$	$\mathrm{The}\;\mathrm{observation}\;\mathrm{probability}\;\mathrm{of}\;c_{i}^{r}$
	${\bar{v}}_{i\to i+1}$	$\mathrm{The}\;\mathrm{average}\;\mathrm{speed}\;\mathrm{from}\;p_{i}$ $\;\mathrm{to}\;p_{i+1}$
	$v_{j}$	$\mathrm{The}\;\mathrm{historical}\;\mathrm{speed}\;\mathrm{of}\;e_{j}$
	$p_{t}\left(c_{i}^{r}\to c_{i+1}^{s}\right)$	$\mathrm{The}\;\mathrm{transition}\;\mathrm{probability}\;\mathrm{from}\;c_{i}^{r}$ $\;\mathrm{to}\;c_{i+1}^{s}$

**Table 2:** Errors between the corrected GPS points and the real GPS points.

Sampling Rate	Mean and Std
60 s	7.24 ± 3.81 (m)
120 s	7.53 ± 4.95 (m)
180 s	8.84 ± 3.42 (m)
240 s	10.25 ± 5.28 (m)
300 s	13.38 ± 6.35 (m)

## Data Availability

Publicly available datasets were analyzed in this study. This data can be found here: [https://www-users.cse.umn.edu/~tianhe/BIGDATA/UrbanCPS/TruckData/TruckData, accessed on 15 March 2022].
